# Science of left ventricular unloading

**DOI:** 10.1177/02676591241268389

**Published:** 2024-07-26

**Authors:** Paolo Meani, Serena Todaro, Giacomo Veronese, Mariusz Kowalewski, Andrea Montisci, Ilaria Protti, Giuseppe Marchese, Christiaan Meuwese, Roberto Lorusso, Federico Pappalardo

**Affiliations:** 1 Department of Cardiothoracic Surgery, Heart and Vascular Centre, 199236 Maastricht University Medical Centre, Maastricht, The Netherlands; 2 Faculty of Health, Medicine and Life Sciences, 5211 Maastricht University, The Netherlands; 3 Thoracic Research Center, Innovative Medical Forum, Collegium Medicum Nicolaus Copernicus University, Bydgoszcz, Poland; 4 Department of Pathophysiology and Transplantation, 9304 Università degli Studi di Milano, Milan, Italy; 5 Anesthesia and Cardiovascular Intensive Care Unit, 9339 Fondazione IRCCS Ca’ Granda Ospedale Maggiore Policlinico, Milan, Italy; 6 Department of Cardiac Surgery, Central Clinical Hospital of the Ministry of Interior, Center of Postgraduate Medical Education, Warsaw, Poland; 7 Cardiothoracic Department, Division of Cardiothoracic Intensive Care, ASST Spedali Civili, Brescia, Italy; 8 Department of Intensive Care and Cardiology, 6993 Erasmus Medical Center, Rotterdam, The Netherlands; 9 Cardiothoracic and Vascular Anesthesia and Intensive Care, 9263 Azienda Ospedaliera Santi Antonio e Biagio e Cesare Arrigo, Alessandria, Italy

**Keywords:** left ventricular unloading, pressure-volume loops, myocardial oxygen consumption, ventriculo-arterial coupling, cardiogenic shock, veno arterial extra-corporeal membrane oxygenation, intra-aortic balloon pump, impella, infarct size, renal unloading

## Abstract

The concept of left ventricular unloading has its foundation in heart physiology. In fact, the left ventricular mechanics and energetics represent the cornerstone of this approach. The novel sophisticated therapies for acute heart failure, particularly mechanical circulatory supports, strongly impact on the mechanical functioning and energy consuption of the heart, ultimately affecting left ventricle loading. Notably, extracorporeal circulatory life support which is implemented for life-threatening conditions, may even overload the left heart, requiring additional unloading strategies. As a consequence, the understanding of ventricular overload, and the associated potential unloading strategies, founds its utility in several aspects of day-by-day clinical practice. Emerging clinical and pre-clinical research on left ventricular unloading and its benefits in heart failure and recovery has been conducted, providing meaningful insights for therapeutical interventions. Here, we review the current knowledge on left ventricular unloading, from physiology and molecular biology to its application in heart failure and recovery.

## Introduction

The heart, throughout the cardiac cycle, is subjected to time-varying forces exerted on the myocardium surface. Borrowing from physics its vocabulary, those forces constitute the “load” of the heart. The energy transferred from an object to displace a force, in this scenario, the heart chambers pumping blood, is defined as work. Since the heart is a biological pump, the energy derives from cellular oxygen consumption. As a result, left ventricular (LV) unloading can be defined as an active reduction in myocardial work, and therefore oxygen consumption, through the application of strategies aimed both at promoting myocardial recovery and reducing V-A-ECMO complications.^1^ This concept needs to be discerned from LV venting, which goal is to indirectly lower the increased LV filling pressures, and the related pulmonary congestion, protecting the lungs and improving gas exchange, with a slight effect on LV work.^1^

A full understanding of the clinical concept of LV unloading relies on detailed knowledge of the underlying physical and physiological bases, as well as the molecular and biological mechanisms related to negative remodeling and myocardial dysfunction.^2,3^ Pressure-volume (PV) loops originally described in the seminal studies of Suga and Sugawa,^4,5^ depicting temporal variations in ventricular volume and pressure on the x- and y- axis, respectively, provide great tool to lay the foundation in the understanding of mechanical and energetic impact of LV overload.^6,7^

When the latter is not adequately managed, specific molecular cascades begin, jeopardizing myocardial recovery.^2,3^

This narrative review elucidates the crucial role of LV unloading in severe cardiogenic shock setting, from the physiological basis to the biomolecular consequences, highlighting the interactions between hemodynamics, metabolism, inflammation, and cellular cycle biology.

## Pressure-volume loops and myocardial energetics

### Analysis of the PV loop

**Figure 1.**
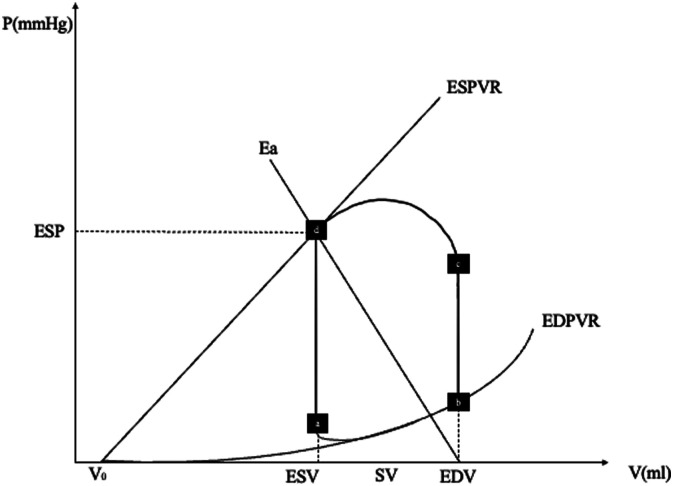
Normal pressure-volume (PV) loop of the left ventricle. On the *x*-axis, ventricular volume. On the *y*-axis, ventricular pressure. See text for details. ESV, end-systolic volume; EDV, end-diastolic volume; SV, stroke volume; EDPVR, end-diastolic pressure-volume relationship; ESPVR, end-systolic pressure-volume relationship; ESP, end-systolic pressure; Ea, arterial elastance.

Analyses of the PV loop are referenced^8,9^. The PV loop can best be described starting at point (a) of Figure 1. At this point, diastole begins, and the blood starts flowing from the left atrium to the left ventricle. In a normal heart, this process occurs at a relatively low ventricular pressure. During a subsequent isovolumetric contraction phase (point (*b)* in Figure 1*),* LV volume remains the same whereas intraventricular pressure increases. When intraventricular pressure exceeds aortic pressure, the aortic valve opens, and blood is ejected into the arterial system (point *c* to *d*) until the end-systolic pressure (ESP) point is reached. Stroke volume, the amount of blood in milliliters (mL) ejected by the LV into the aorta at each cardiac cycle is defined by V_ED_ – V_ES_, where V_ED_ = end-diastolic volume and V_ES_ = end-systolic volume. At the end of the ejection phase, the aortic valve closes, and the isovolumetric relaxation phase begins. At point *a*, the mitral valve opens and ventricular filling restarts.

### Preload and afterload

Preload is indexed by the end diastolic volume or pressure, even though the relationship between filling pressure and filling volume varies in disease states involving diastolic dysfunction.^6,7^ Preload variations lead to a displacement of the PV loop rightward or leftward, accordingly to a preload increase or decrease. This changes in preload, in the experimental context, can be obtained through inferior vena cava occlusion reducing the amount of blood returning to the heart, hence causing the PV loop to progressively shift leftwards, indicating a lower end-diastolic volume.^10^

The concept of “effective arterial elastance (Ea)”, namely the arterial resistance to dilatation, according to Sunagawa et al.,^11,12^ represents afterload incorporating all extracardiac factors impending ventricular ejection. Ea complex equation derives from the Windkessel model, however, the slope of the Ea line con be approximated as following (for better understanding, refer to Figure 1):

∆P=CO x TPR

$$P_{A}-P_{V}=HR\;x\;SV\;x\;TPR$$

where P_V_ can be approximated to zero, and P_A_ to MAP and therefore ESP, resulting in

ESP=HR x SV x TPR

$$\frac{ESP}{SV}=HR\;x\;TPR$$

Observing Figure 1 can be inferred that ESP to SV ratio equals Ea, thus

$$E_{a}=HR\;x\;TPR$$

where CO – cardiac output, TPR – total peripheral resistance, HR – heart rate, SV – stroke volume, ESP – end systolic pressure^2^:

An increase in afterload (i.e. increase in TPR and/or increase in heart rate) leads to Ea slope rise along the ESPVR, and, then, the PVL shifts rightward (if ESPVR remains constant), associated with a ESP rise and SV drop. According to Walley et al.,^7^ we can state that ventricular elastance (i.e., the change of pressure over volume, dP/dV) interacts with the arterial elastance during the ejection phase. Basically, an increased afterload determines an ESP increase at the expense of ejection, which negatively impacts on the heart mechanical efficiency.

### End-systolic pressure-volume relationship (ESPVR)

The ESPVR is approximately linear in the physiological range of end-systolic pressures (P_es_) and volumes (V_es_). It is expressed as: P_es_ = E_es max_ x (V_es_ – V_0_), where E_es max_ is the slope of the ESPVR, V_0_ is the intercept of this line with the *x*-axis.^13^ Of note, the physiological relevance of its volume-axis intercept, V0, remains unclear since it might strongly be influenced by either the method of extrapolation (linear vs logarithmic) or the inotropic state.^14^

The term E_es max_ (maximum ventricular end-systolic elastance) represents a load-independent measure of ventricular contractile state. The latter rises with positive inotropism (i.e. dobutamine, milrinone, levosimendan) and sympathetic activation; on the contrary, E_es max_ decreases with negative inotropism (i.e. calcium channel blockers and β-blockers), myocardial ischemia or infarction.^8,9^

As the slope of ESPVR has the dimensions of elastance (dP/dV), the entire cardiac cycle can be interpreted as a cycling variation of elastance. In other words: “the time-varying elastance model consists of a single elastic element whose elastance is time-varying. This elastance increases gradually with time during systole and is maximized at the end of systole”.^15^

### End-diastolic pressure-volume relationship (EDPVR)

Conversely to ESPVR, the EDPVR is non-linear and represents the lower boundary of the PV loop. The EDPVR reflects the passive mechanical properties of the LV chamber which are highly influenced by the size, orientation and mass of myocytes, as well as the extracellular matrix. As a consequence, ischaemia, oedema, remodeling, fibrosis, and hypertrophy affect the EDPVR. Its slope (dP/dV) indexes LV chamber stiffness and is load-dependent. Compliance is the mathematical inverse of stiffness (i.e. dV/dP) and, according to Walley,^7^ can be described as:

P=S×log(Vm−V)/(Vm−Vo)

where S represents diastolic myocardial stiffness, Vm is the maximum allowed diastolic ventricular volume (these limitations are determined by the pericardium, myocardiocytes’ cytoskeleton, extracellular matrix and junction proteins), and Vo is the diastolic volume at zero pressure. The curvilinear EDPVR explains the loss of compliance at high volumes, namely when LV is progressively dilated.

### Myocardial work and oxygen consumption

**Figure 2.**
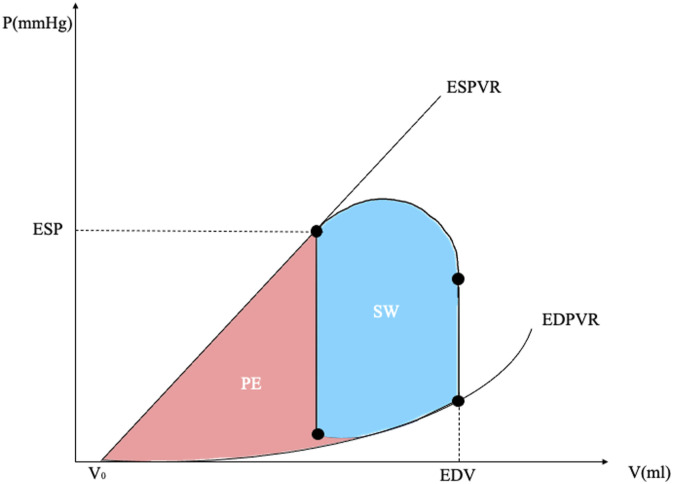
Left ventricular energetics. On the *x*-axis, ventricular volume. On the *y*-axis, ventricular pressure. See text for details. SW, stroke work; PE, potential energy; ESV, end-systolic volume; EDV, end-diastolic volume; EDPVR, end-diastolic pressure-volume relationship; ESPVR, end-systolic pressure-volume relationship; ESP, end-systolic pressure.

The area of the PV loop corresponds to the external myocardial work, that is the work performed by the left ventricle to eject blood into the aorta. However, for the determination of the myocardial oxygen consumption (MVO_2_), the additional area under the ESPVR must be taken into account, representing the potential energy (PE) stored into the ventricular wall, related to the viscoelastic properties of the myocardium (see Figure 2).^16^ Therefore, stroke work (SW) and PE can be mathematically expressed as:

- SW = $\int_{Ved}^{Ves}P\left(V\right)dV$ - $\int_{Ved}^{Ves}EDPVR\left(V\right)dV$ where Ved and Ves are end-diastolic and end-systolic volumes, representing variations in volume during the cardiac cycle.
- PE = $\int_{V0}^{V1}ESPVRdV$ - $\int_{V0}^{V1}EDPVR\left(V\right)dV$ where V0 is the intercept of EDPVR with the *x*-axis.

The sum between SW and PE is defined pressure-volume area (PVA = SW + PE, see Figure 2). It has been experimentally demonstrated that MVO_2_ is linearly related to PVA^17^ and not SW alone, as energy expenditure is related both to internal and external work.

It is important to stress that, alongside previously depicted elements influencing myocardial work, since a single PV loop corresponds to a single heartbeat, higher the heart rate, higher the energy expenditure and therefore oxygen consumption per minute.

### Cardiac mechanical efficiency

As a matter of fact, the energy produced by the heart is partially converted to external power, related to the production of cardiac output,^18^ whereas the rest gets “lost” as internal power (potential energy). The mechanical efficiency of the heart can be expresses as: $E=\frac{SW}{MVO2}\;\frac{\left[J\right]}{\left[mLO_{2}\right]}$ .

Under normal conditions this ratio is ≈ 10%–25%. The residual energy is mainly dissipated as heat.^19^ Katz et al. demonstrated that heart rate and the maintenance of a given wall stress are the most expensive components of MVO_2_.^20^ The LV volume overload (i.e. dilated cardiomyopathy or secondary to acute LV failure), as well as heart rate increase (as a compensatory mechanism in acute LV disfunction) significantly rise the MVO_2_. The latter, can be derived from the product of coronary sinus blood flow (CS_bf_) and the arterial (CaO2) – coronary sinus oxygen content (CcsO2) difference: MVO_2_ = CS_bf_ x (CaO2 – CcsO2), where CaO2 is arterial oxygen content that equals coronary oxygen content.^21^ Obviously, conditions which significantly drop the coronary flow negatively impacts on oxygen consumption and delivery balance.^22^

### Ventriculo-arterial coupling

Ventriculo-arterial coupling indicates the dynamic interaction between the ventricular pump ejection and the related change in arterial pressure.^23^ Therefore, ventriculo-arterial coupling depicts the global cardiovascular performance and efficiency, describing the relationship between the ventricle contractile function (either the left or the right ventricle) and the arterial load during each cardiac cycle.^24^

This concept is mathematically expressed as the Ea/Ees ratio.^13^ Ees is a load-independent myocardial contractility index and systolic stiffness,^25^ while Ea incorporates several elements associated with arterial load, which include peripheral vascular resistance, total arterial compliance, impedance, and systolic and diastolic time intervals. An optimal ventriculo-arterial coupling (i.e., when the Ea/Es approximates 1) allows an optimal ratio of useful work (external work, SW) to PVA (total work, including wasted energy).^7^ The ventriculo-arterial de-coupling has been emerged in different clinical settings, such as acute heart failure cardiogenic shock,^23^ and even septic shock.^24^ Likewise, the specular concept can be applied to the right ventricle which is called right ventricle-pulmonary artery coupling.^26^

### Ventricular interdependence

The ventricular interaction occurs both during both systole and diastole. The main determinants of diastolic interaction are the interventricular septum displacement, following by ventricle compliance and geometry alterations, and the pericardium.

The systolic interdependence consists of LV contraction contribution to pressure and stroke volume development by the right ventricle.^27^

### PV loop during cardiogenic shock

**Figure 3.**
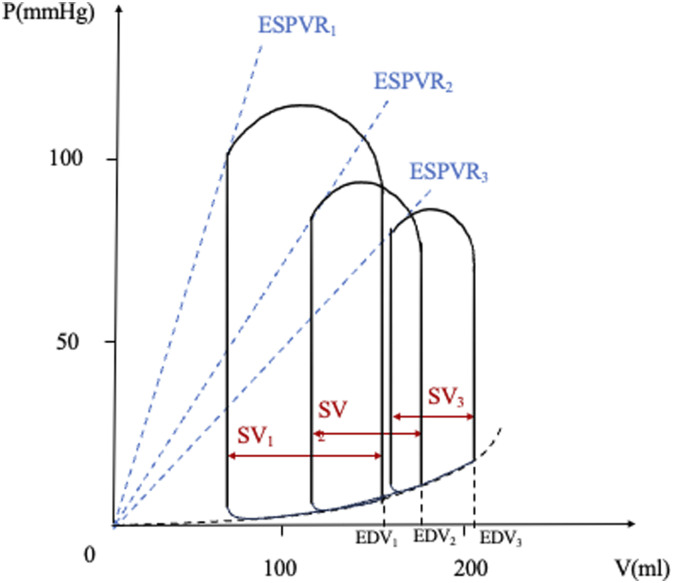
Pressure-volume loop during cardiogenic shock. The change of the slope of the end-systolic pressure-volume relationship (ESPVR) line from 1 to 3 and its rightward shift indicate a reduction of LV contractility, associated with a reduction of the stroke volume and with an increase of left ventricular end-diastolic pressure. SV, stroke volume; EDV, end-diastolic volume; EDPVR, end-diastolic pressure-volume relationship; ESPVR, end-systolic pressure-volume relationship. Adapted from: *Nir Uriel et al, Mechanical unloading in heart failure. J Am Coll Cardiol. 2018 Jul 31;72(5):569-580.*

The following changes occur in the PV loop analysis during cardiogenic shock^28^ (see Figure 3):

1. The ESPVR lowers, mirroring a reduction in inotropism of the LV.
2. The PV loop shifts downward and rightward.
3. ESP decreases, also related to an impaired LV contractility.
4. EDP and EDV increase as a compensatory mechanism to maintain SV. Later on, as cardiogenic shock progresses, these mechanisms begin to fail, with a subsequent reduction in SV.
5. Blood pressure (the height of the ESP point) decreases, leading to a reduction in tissue perfusion pressure.

### Unloading and reduction of myocardial oxygen consumption

**Figure 4.**
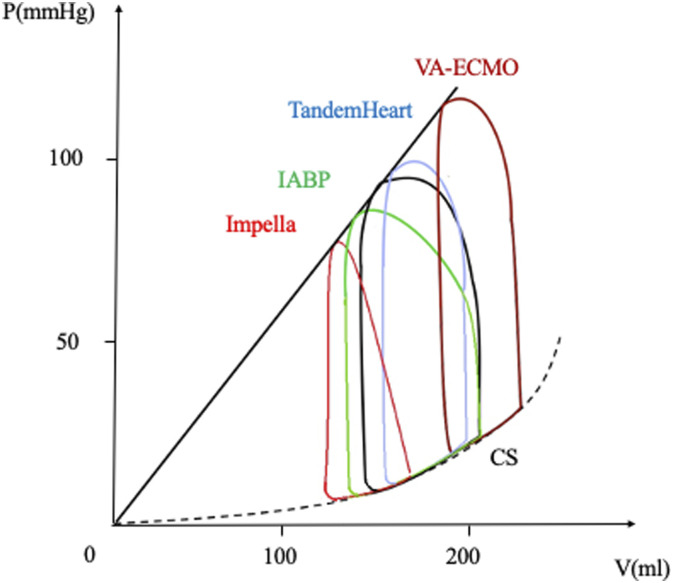
Unloading during cardiogenic shock. V-A-ECMO leads to an upward and rightward shift of the PV loop due to an increased afterload (dark red curve). The combined application of LV unloading techniques, such as TandemHeart (blu curve), IABP (green curve) or Impella (red curve), determine a leftward shift of the PV loop. See text for details. IABP, intra-aortic balloon pump; CS, cardiogenic shock; V-A-ECMO, veno-arterial extra-corporeal membrane oxygenator. Adapted from: *Donker, DW, et al., Left ventricular unloading during veno-arterial ECMO: a review of percutaneous and surgical unloading interventions. Perfusion. 2019 Mar;34(2):98-105;* and *Kapur, NK, et al., From bedside to bench and back again: translational studies of mechanical unloading of the left ventricle to promote recovery after acute myocardial infarction. F1000Res. 2018 Nov 27;7:F1000 Faculty Rev-1852.*

On one hand, unloading is defined as the reduction of the left ventricular myocardial work by minimizing myocardial oxygen consumption, reducing hemodynamic forces that lead to ventricular remodelling. On the other hand, in terms of the pressure-volume relationship, unloading is considered the reduction in PVA area, strictly correlated to myocardial oxygen consumption, as previously described. Given all these premises, the study of PV loops following different strategies of mechanical support highlights several findings^6,25,29,30^ (see Figure 4).

### Limits and downsides of the PV loop

The PV loop analysis must be considered as a useful tool to understand the physiopathology behind heart functioning and malfunctioning. Nonetheless, it has several limits lying in the physical concept itself of the PV loop. Foremost, the PV loop is hardly obtainable in a clinical setting, both due to costs of the equipment required and the invasiveness of the technique itself. In addition, the sole analysis of the loop does not take into account a variety of other key elements necessary to grasp cardiac pathology in its entirety, such as myocardium wall thickness, cross fiber shortening phenomenon.

## Unloading effects related to mechanical circulatory supports

### Intra-aortic balloon pump (IABP)

IABP is pump which deflates in systole and inflates during diastole. This leads to reduce central aortic pressure, aortic impedance, systemic vascular resistances as well as raising central aortic mean diastolic pressure and lowering central aortic end-diastolic pressure. Additionally, IABP increases coronary blood flow, due to increased diastolic perfusion gradient.^31^

Therefore, IABP ultimately increases stroke volume and ejection fraction.^8,32^ Despite improved LV ejection, myocardial stroke work decreases as a consequence of reduced ESP, leading to reduce also PVA^33^ (see Figures 2 and 4). As a consequence, IABP improves ventriculo-arterial coupling.^34^

### Impella

Among the strategies aiming to percutaneously unload the LV, Impella, a left ventricular assistant device, is the most promising technique.^35^ After positioning of the Impella device the characteristics of the PV loop change (see Figure 4):

(1) the PV loop shifts leftward, meaning lower LV volumes and pressures during a cardiac cycle.
(2) the PV loop assumes a unique triangular shape, due to the loss of isovolumic contraction and relaxation phases, secondary to the continuous pumping of the blood from the LV to the aorta.
(4) the PVA is reduced, therefore the MVO_2_ is reduced. This phenomenon has been experimentally demonstrated by Braunwald who showed that, in a canine model, the most important determinant of MVO_2_ is the tension-time index, expressed as the area under the left intraventricular pressure curve.^36^

Nevertheless, Impella requires skilled and trained staff since this device might be lead to an increased risk of complications, such as major bleeding, stroke, ventricle perforation, access-site related infection and bleeding and lower limb ischemia.^37^

### TandemHeart

Another mechanical circulatory support which is able to actively unload the LV is TandemHeart (Cardiac Assist, Inc., Pittsburgh, PA). Blood is drawn from the LA and returns to a main artery, such as subclavian, axillary or femoral arteries. Under LA-to-arterial support a reduction in end diastolic pressure and LV stroke volume is observed, while end systolic volume increases to obtain enough LV pressure generation in order to eject blood in the aorta. These hemodynamic changes can result in a decreased PVA and concomitant MVO_2_ reduction^6^ (see Figure 4).

### LV overload due to veno-arterial ECMO (extra-corporeal membrane oxygenation)

Veno-arterial ECMO (V-A ECMO) primarily alters the equilibrium between the arterial and venous circulation, by draining blood from the venous compartment and pumping into the arterial system. First of all, V-A ECMO reinfuses oxygen blood pressurizing the aorta, which may rise LV afterload, as well as LV preload, left atrial and pulmonary pressure, even if indirectly. Secondly, the venous drainage reduces the right ventricle preload, the right ventricle output and therefore the pulmonary circulation. Moreover, the native and artificial blood pumps act independently, linked by the central venous, pulmonary, and arterial circulations. Therefore, as a results, the patient-device interplay is very intricated.^38^ Depending on this complex interaction, the retrograde ECMO flow can directly increase LV afterload. Besides the Anrep effect,^38^ the Starling mechanism is the only way for the LV to overcome the increased afterload. The blood consequently accumulates in the LV and the LVEDP rises, as well as the pulmonary capillary wedge pressure. The PV loop becomes narrow (native LV stroke volume drop) and taller (increased afterload pressure), and shifts rightward and upward along the EDPVR (see Figure 4).^6^ MVO_2_ under ECMO might therefore be increased.^6^ Although the hemodynamic impact of central cannulation is still matter of debate,^38,39^ this phenomenon occurs more often in the peripheral configuration.^38^

The application of conservative, either pharmacological or non-pharmacological measures (such as avoiding vasopressors, vasodilators use, diuretic administration or continuous renal replacement therapy, positive end-expiratory pressure ventilation, limiting ECMO flow) can mitigate or prevent the aforementioned rightward and upward PV loop shift.

When a non-invasive strategy is not able to prevent this ECMO shortcoming, the combined application of ECMO and other mechanical support, such as IABP or Impella has shown a downward and leftward shift of the PV loop^40^ suggesting a subsequent reduction in MVO_2_.

LV overload might lead to aortic-valve non-opening, intra-cavity blood stasis and eventually danger intraventricular thrombosis.^41^ Furthermore, LV overload significantly jeopardize myocardial recovery.^42^ Therefore, different techniques aiming to unload the LV, either surgical or percutaneous, has been developing.^43^

### LV unloading strategies during veno-arterial ECMO

Among the LV unloading strategies, the trans-aortic percutaneous left ventricular assist device, Impella, in combination with V-A ECMO, effectively mitigates the progression or even avoids the overload of the LV.^29^ This configuration is often called ECPELLA or ECMELLA. The hemodynamic improvement due to Impella implementation during V-A ECMO support are summarized as follows: (1) increasing cardiac power output, (2) increasing oxygen supply (if the blood oxygenation is not compromised by a severe pulmonary oedema), and (3) decreasing oxygen demand.^29^ By continuous pumping of blood from the LV to the aorta, the PV isovolumic periods are lost. As a consequence, the PV loop shifts from its normal trapezoidal shape to a triangular shape. Then, LV is progressively unloaded, moving leftward.^44^

Schrage et al retrospectively enrolled over 600 patients with cardiogenic shock treated with either V-A ECMO + IMPELLA or V-A ECMO alone. The results showed a lower 30-day mortality in the ECMELLA group, in spite of increased complication rate observed.^45^ Further analysis carried on by Schrage et al indicate a reduction in mortality solely when early unloading is performed, whereas late unloading isn’t associated with improved survival rates.^46^

**Figure 5.**
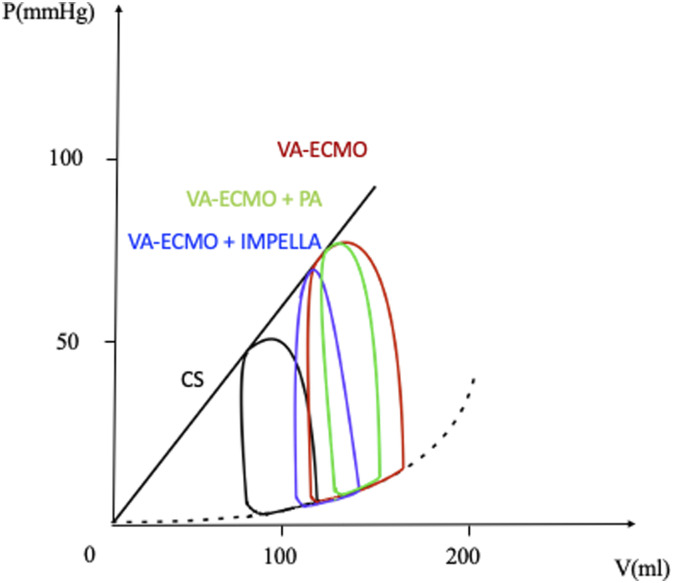
Unloading under V-A-ECMO. Combined application of V-A-ECMO and IMPELLA (blue curve) or pulmonary artery cannula (green curve) as unloading techniques. CS (black curve), cardiogenic shock; PA, pulmonary artery; V-A-ECMO, veno-arterial extracorporeal membrane oxygenator. Adapted from: *Meani, P., et al., Transaortic or Pulmonary Artery Drainage for Left Ventricular Unloading in Venoarterial Extracorporeal Life Support: A Porcine Cardiogenic Shock Model. Semin Thorac Cardiovasc Surg, 2021. 33(3): p. 724-732. and Mlcek, M., et al., Atrial Septostomy for Left Ventricular Unloading During Extracorporeal Membrane Oxygenation for Cardiogenic Shock: Animal Model. JACC Cardiovasc Interv, 2021. 14(24): p. 2698-2707.*

Different grades of unloading- namely PV leftward shift- can be achieved with other percutaneous strategies, such as IABP, atrial septostomy, pulmonary artery drainage cannula (see Figure 5). Of note, ECPELLA provided the most effective PVA reduction compared to atrial septostomy and pulmonary artery drainage cannula in a cardiogenic shock porcine model.^47,48^ Although an attempt to standardise techniques has been done, at the moment there are no clinical data supporting an unloading strategy over the other.^39^ Furthermore, to the best of our knowledge no RCT has been published comparing different unloading strategies, nor indicating differential indication for their use.^47^

## The biological response to ventricular unloading

The aforementioned hemodynamic and energetic mechanisms beyond LV unloading translate into a biological response, which consists of several pathways (such as cell hypertrophy-atrophy, calcium handling, cytoskeletal, protein and gene expression, neurohormones, inflammation, extracellular matrix and fibrosis, endothelium and vasculature, angiogenesis, cardiac metabolism and bioenergetics^49^). On one hand, the impact of LV pressure and volume overloads is intimately related to the setting when those occure (i.e. acute or chronic). On the other hand, the effect of LV unloading is also ultimately influenced by the underlying myocardial etiology (namely ischemic or non-ischemic disease).

### Chronic non-ischemic and ischemic setting

#### Molecular biology and genetic/epigenetic determinants of unloading response

LV unloading showed beneficial molecular and genetic/epigenetic response in chronic ischemic and non-ischemic heart. First of all, LV pressure and volume overload alters cardiac energy metabolism. Changes are observed in substrate utilization, oxidative phosphorylation and adenosine tri-phosphate (ATP) transfer and utilization. When HF occurs, a reduced free fatty acid utilization and glycolysis leads to a reduction in energetic substrate. In addition, an impaired oxidative phosphorylation chain leads to the increase of uncoupling proteins and insufficient supply of ATP.^50^

Mechanical unloading could potentially limit, or reverse, those metabolic adaptation, activating pathways of cardio-protection and recovery.

Diakos et al. investigated how left ventricular assist device (LVAD) mechanical unloading of the heart induced metabolic changes in the myocardium. Thirty-one patients with advanced HF undergoing LVAD as a bridge to transplantation were included and myocardial tissue samples were collected before and after LVAD support. The study demonstrated an up-regulation of glycogenolysis, glycogen utilization, and decrease in intermediates of Krebs cycle post-LVAD implantation. These alterations suggest a glycolysis-mitochondrial oxidation mismatch. However, increased levels of amino acids were registered after LVAD unloading. This could potentially act as a compensatory mechanism where amino acids enter the tricarboxylic acid cycle through anaplerosis, a group of reactions targeted to replenish these intermediates, contributing as an alternative energy source.^51^

Furthermore, Anisha et al compared failing hearts before and after LVAD implantation, identifying improvements in metabolic and transcriptional defects occurring during HF. Levels of acylcarnitines, products of glucose, amino and fatty acid oxidation, generally reduced during HF, significantly increased after LV unloading. Analogue results were shown for organic acids and amino acids.^52^

Likewise, transcription gene defects were nearly all reversed after LVAD unloading. Transcripts encoding proteins belonging to metabolic pathways and mitochondrial enzymes were analyzed: messenger ribonucleic acid (mRNA) levels of peroxisome proliferator-activated receptor γ coactivator 1α (PGC1A), a regulator of mitochondrial metabolism, and of enzymes operating in the free fatty acid oxidation chain were strongly reduced in HF. Even transcripts of enzymes in pyruvate metabolism and mitochondrial electron transport chain were decreased. After mechanical unloading all transcription factors mentioned were upregulated and globally increased, confirming changes observed in protein levels. Authors conclude that these data indicate a global improvement of substrate utilization and energy production, mediated by LVAD unloading.^50^

Beyond modification of cardiac energy metabolism, pressure and volume overload induces a pathological remodeling of the LV, consisting of myocardial hypertrophy, tissue fibrosis and changes in cellular autophagy. Keith et Al examined cardiac remodeling through an experimental setup of pressure overload and unloading in a mice HF model. Pressure overload was induced by aortic surgical constriction, while aortic de-banding simulated unloading. Study results demonstrated an increase in LV mass and strain, thickening of LV wall, myocardial hypertrophy and fibrosis 4 weeks after aortic bending. These pathological changes were accompanied by a fibroblast growth, collagen deposition, cardiomyocyte hypertrophy and higher matrix metalloproteinases (MMPs) and tissue inhibitors of metalloproteinases (TIMPs) isoform mRNA expression. After 2 weeks of pressure unloading, a significant reduction in these alterations was observed, indicating potential reversibility of remodeling mechanisms.^53^

Dysregulation of energy metabolism, apoptosis pathways and structural modification are known to be present also in post-ischemic hearts. In myocardial ischemia several chemokines are known to be up regulated, mediating apoptosis and tissue remodeling. Stromal derived factor 1 alfa (SDF-1𝛼) is a chemokine that acts as homing system in hypoxic conditions, directing stem cells towards hypoxic tissue.^54^

Although “time is tissue” is used as main paradigm in acute myocardial infarction management, up to 25% of patients after acute myocardial infarction (AMI) develop HF.^55^ Esposito et al speculated that this poor outcome is related to ischemia-reperfusion injury and could potentially be reversed by cardioprotective strategies, such as primary unloading. The experimental setup consisted of swine undergoing induced ischemia treated with a combination of reperfusion alone, unloading after reperfusion and unloading before reperfusion. Results showed that LV unloading 30 min before reperfusion reduced down-regulation of genes associated with energetic metabolism and increased mRNA of genes belonging to the respiratory chain. Analysis on SDF1-𝛼 highlight how primary unloading may preserve SDF1-𝛼 levels, inducing cardio-protection. LV unloading was also demonstrated to reduce pro-apoptotic signaling and infarct size, ultimately potentially improving AMI outcomes.^56^

### Acute ischemic setting

#### Timing and infarct size

LV mechanical unloading, during the acute phase of myocardial infarction (AMI), reduces infarct size (IS).^52,53^ IS represents a crucial endpoint in clinical trials enrolling patients with AMI. It can be estimated through three different methods: 1. By dosing serum biomarkers 2. By performing technetium (Tc)-99 m sestamibi single-photon emission computed tomography myocardial perfusion imaging 3. By performing magnetic resonance imaging.^54^

It was demonstrated that IS (per 5% increase) is strongly associated with all causes mortality (hazard ratio 1.19) and hospitalization due to HF (hazard ratio 1.20) within 1 year from the event, in patients without prior myocardial infarction.^55^

The reduction of IS seems to be linked to a MVO2 decrease that in turn reduces the oxygen supply-demand imbalance. By lowering LV end-diastolic pressure and increasing LV end-systolic elastance, LV unloading decreases the stroke work, which represents one of the main determinants of MVO2.^6,56^

Several studies were conducted in canine models. For instance, Saku K. et al showed that the IS reduction was much more effective using total support with Impella device rather than partial support or no support (control, 16.3 ± 2.6; partial support, 8.5 ± 4.3; and total support, 2.1 ± 1.6%).^52^ Saku K. itself et al reached similar findings using total LVAD support which dropped MVO2 and IS, whereas partial LVAD provided less MVO2 reduction and subsequently larger IS. In partial LVAD, since the LV keeps on ejecting, the LVAD therefore decreases LV end-diastolic volume but simultaneously rising mean arterial pressure, which ultimately increases end-systolic volume.^56^ On the other hand, in total LVAD, the LV no longer ejects because LV pressure drops down under the mean arterial pressure due to low LV preload.^56^ Furthermore, Sunagawa et al. experimented the “mechano-chronotropic unloading” adding a bradycardic agent such as ivabradine to the unloading of LV by Impella and demonstrated a strong synergic action in reducing IS (control 56.3 ± 6.5, Impella 39.9 ± 7.4 and Impella + Ivabradine 23.7 ± 10.6%).^53^

Furthermore, also LV unloading timing in AMI setting seems to play a crucial role. Kapur et al demonstrated that LV unloading could delay coronary revascularization during the acute phase of myocardial infarction. In a swine model of ischemia/reperfusion the duration of ischemia in the control group was 120 min, in the treatment group was 120 min + 30 min with LV unload achieved by left-atrial to femoral artery bypass system. It was proven that LV unloading not only reduced wall stress (44,658 vs 22,963 dynes/cm2), SW (2823 vs 655 mmHg·mL) and IS (49% vs 28%) but also increased phosphorylation of reperfusion injury salvage kinase pathway proteins from non-infarcted LV tissue, showing to be protective from reperfusion injury.^6^ In addition, recent preclinical data on swine model show that primary unloading of the LV in AMI, despite delaying reperfusion, reduces infarct size (IS) when compared to primary reperfusion (73 ± 13% vs 42 ± 8%; *p* = 0.005)^57^; (33.3 ± 5% vs 62.2 ± 1.7% infarct/area-at-risk, *p* < 0.01)^56^ and when compared to continued occlusion (52 ± 15% vs 34 ± 6%, *p* = 0.03), whereas ECMO did not.^58^ Moreover, improvements of LV scar size and cardiac function were noted also 28 days after AMI.^59^

This may be due not only to myocardial oxygen demand reduction, but rather may involve activation of a “myocardial protection program” introducing a new concept of “mechanically conditioning” which ultimately could decrease HF deriving from myocardial damage.

One of the mechanisms involved is the activation of a protective cytokine cascade that brings to reduction of ischemia-reperfusion injury. In fact, an increased production of SDF-1and its receptor CXCR4, within the infarct area, was noted by Kapur et al. in swine ischemia-reperfusion models. These two mediators are involved in ischemic pre-conditioning-mediated myocardial salvage by activating the extracellular signal-regulated kinases (ERK) -protein kinase b (akt) and inhibiting glycogen synthase kinase-3b. Moreover, the authors noted a down-regulation of genes associated with mitochondrial function and cellular respiration and a reduction in cellular apoptosis signaling.^59,60^

Another interesting finding is that, by reducing SW, LV unloading in AMI may partially attenuate myocardial ischemia by providing increased microcirculatory collateral blood flow to myocardium at risk. Briceno et al. documented an increased pressure-derived coronary flow index (CFI) to the ischemic myocardium which is ultimately associated with smaller infarct size. Compared to Impella, ECMO did not show any improvement on SW, CFI or infarct size.^61^

Certainly, further studies in humans are needed. Nowadays LV unloading is only indicated for emergent high-risk percutaneous coronary intervention (HR-PCI) in case of LV disfunction. It is though not indicated for STEMI without cardiogenic shock and delaying of reperfusion is not recommended.

The first attempt of extending this indication is the on-going “door-to-unloading DTU STEMI trial” which randomly assigns patients with acute ST-elevation myocardial infarction to receive either LV unloading by Impella CP followed by immediate reperfusion or delayed (30 min) reperfusion. In a pilot study of 50 patients, the investigators did not report safety signals that would have precluded proceeding to a larger pivotal study.^60^ The feasibility of delaying primary reperfusion opens to the possibility of studying even other interventions to reduce ischemia-reperfusion injury that nowadays has not a targeted therapy.

#### Myocardial recovery

LV unloading not only reduces IS, but it may possibly help myocardial recovery.^3,62^

Studies conducted on human hearts explanted from transplantation recipients, after a period of mechanical circulatory support with LVADs, showed that the end-diastolic pressure–volume relationship of human end-stage failing hearts can shift back to normal values, introducing the concept of “reverse remodeling”, overcoming the traditional idea that myocardial remodeling is irreversible. However subsequent studies showed that the molecular pattern associated with HF remains in the reverse-remodeled myocardium despite apparent normalization. In contrast, true myocardial recovery consists in regaining both normal function and molecular makeup.^63^

The mechanisms which bring to cardiac inflammation and fibrosis are still unclear. Several actors have been part of the process.

Integrins, for instance, are cell surface receptors which act in both cellular adhesion and signaling. In the myocardium they are recognized as mechano-transducers. In addition to their direct effects on cellular proliferation, migration and survival, mediated by their binding to extra-cellular matrix proteins, integrins can potentiate signals from soluble growth factors such as transforming growth factor β1 (TGFβ1), and act as receptors for matricellular proteins. It is apparent that increased integrin expression in specific cell types, may be linked to fibrosis.^64^

Another key element in myocardial remodeling are fibroblasts. Mechanical stretch occurring in HF induces activation of fibroblasts and is capable of stimulating production of extracellular matrix and moreover of up-regulating chemokine production and triggering typical inflammatory pathways in human cardiac fibroblast cell cultures. Fibroblasts also appear to be capable of activating inflammatory cells and inducing further recruitment of monocytes by allowing trans-endothelial migration into the cardiac tissue.^65^

Moreover, HF not only induces inflammation in the heart but can lead to peripheral tissues disfunction (bone marrow, spleen, gut, adipose tissue) due to both direct (inflammatory) and indirect (hemodynamic) mechanisms. It is thus clear that inflammation and HF are strictly connected and mutually reinforce each other.^66^

In this regard, acute myocarditis represents a model in which inflammation and HF strictly coexist and usually its treatment requires the use of LV Impella alone, in combination with extra-corporeal life support (ECLS) (ECPELLA), or in combination with right ventricle Impella RP (BIPELLA) in case of biventricular failure. The recent multicentered randomized DanGer Shock trial randomized 360 patients with STEMI and cardiogenic shock to either microaxial flow pump (Impella CP device) or standard treatment; all patients enrolled underwent a revascularization procedure.^67^ This trial showed a lower risk of death at 180 days among those who underwent circulatory support with a microaxial flow pump; although this group had a higher incidence of adverse events, such as bleeding or sepsis, these complications did not overshadow the benefits of Impella CP, in terms of overall survival. The authors suggest LV unloading and subsequent reduction of oxygen consumption as one of the possible explanations for the higher survival rate in the intervention group.

The use of ECLS without unloading strategies, particularly in myocarditis, leads to an increase in myocardial stress which brings to activation of cardiac mechano-transduction pathways and, over time, induces inflammatory reactions. Myocardial abnormalities include immune cell infiltration, cardiac fibrosis, dysregulation of titin function and impaired energy metabolism. The use of prolonged Impella support (PROPELLA) may reverse this remodeling process. Tschope et al. reported a case in which LV Impella support was used, through axillary artery, for 39 days in awake and mobilized patient, together with standard HF and immunosuppressive therapy, and demonstrated reduction of myocardial inflammation, modulation of cardiac remodeling, and restoration of physiological metabolic machinery.^62^

**Figure 6.**
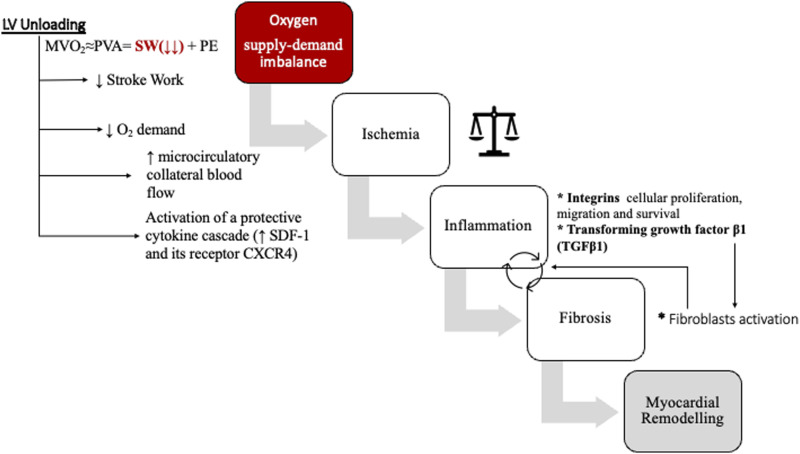
Effects of LV unloading on infarct size and myocardial remodelling. Increased afterload leads to higher myocardial workload, causing an oxygen supply-demand imbalance. The result is tissue ischemia, followed by the activation of the inflammation cascade, fibrosis and ultimately myocardial remodelling. LV unloading, reducing stroke work and therefore oxygen consumption, may interfere in these pathological mechanisms. See text for details.

In conclusion the use of LV unloading strategies can preserve myocardial damage by reducing oxygen supply-demand imbalance in myocardial infarction and could possibly prevent, and even revert, the inflammatory cascade which brings to HF and myocardial remodeling (see Figure 6).

## Besides the heart, renal benefits

There is a paucity of data surrounding outcomes in cardiogenic shock patients with concurrent acute kidney injury (AKI); undoubtedly, cardiogenic shock complicated by AKI requiring renal replacement therapy is associated with increased mortality.^68^

AKI in cardiogenic shock has been postulated to be caused by venous congestion and reduced renal perfusion.^69^

Protection against AKI has been reported with the use of Impella during HR-PCI.^70^

Mehran and colleagues developed a well-validated and widely accepted risk score that could be readily applied by clinicians to evaluate individually predicted patient risk for developing AKI following PCI.^71^ The first signal of the potential role of partial hemodynamic support with Impella in preventing AKI in the HR-PCI cohort was detected in the PROTECT II trial. This finding was subsequently reinforced with a randomized controlled trial published by Flaherty et al in 2017 demonstrating that Impella 2.5 reduced the incidence of AKI to 5.2% compared to 27.8% in the unsupported control group. Patients at high and very high risk of AKI (Mehran risk scores 11–16 and ≥16) also experienced significant protection from AKI (9.3% observed AKI incidence vs 26.1% predicted (64.4% lower risk, *p* = 0.0004) and 28.6% versus 57.3% (50.1% lower risk, *p* = 0.008, respectively). It is also noteworthy that AKI in the Impella-supported arm was exclusively stage 1 (≥0.3 mg/dL absolute or 1.5- to 2.0-fold relative increase in serum creatinine), and there were no patients who required hemodialysis, despite a predicted dialysis rate of 12.6% in the highest risk cohort. Lastly, increasing contrast load was surprisingly not shown to have a significant effect on the development of AKI when Impella was used.^59^

The authors can only speculate as to the mechanism of these renal protective effects: (1) the salubrious effect of continuous unperturbed renal perfusion and (2) increase in the rate of intraprocedural contrast washout from the renal tubules, thus diminishing contrast-related nephrotoxic effects.^65^

On the contrary, the use of the IABP significantly increased the risk of AKI.^61,72^

Existing data indicate key differences between ECMO and Impella support on renal function that may mediate this effect either by themselves or (more likely) in combination with one another: hemodynamic considerations, effects on atrial natriuretic peptide (ANP) production, and a differential inflammatory response.

Both devices increase perfusion pressure to a similar extent, but the devices have fundamentally different effects on LV preload and afterload. This may potentially alter downstream ANP production: ANP is produced by atrial myocytes in response to myocardial stretch resulting from volume and pressure overload. In an effort to decrease blood volume and relieve atrial stretch, ANP release increases sodium and water excretion. Importantly, ANP also inhibits the renin-angiotensin-aldosterone system (RAAS): RAAS-dependent signaling is a principal player in preventing low GFR. Reflecting this atrial stretch, serum ANP is increased after V-A-ECMO treatment.^73^

Normally, ANP signaling ceases upon the alleviation of atrial stretch (resulting from natriuresis). Yet, in the setting of V-A-ECMO, afterload is artificially preserved leading to elevated atrial pressure. This would maintain ANP-dependent signaling and inhibition of the RAAS. In this respect, Impella support is fundamentally different. Due to its position, the Impella directly unloads the LV, and upstream unloading of the left atrium is observed.^74^ Therefore, atrial stretch is reduced during Impella support, and ANP/RAAS signaling would be normalized.

This important distinction between these devices is theoretically exploited in the clinical setting of ECPella, reversing the atrial response to distension and increasing the total cardiac output by the simple adding of the two pumps flow.

## Future perspectives

The echocardiographic evaluation including 2D and 3D cavity volume measurements and Doppler-based parameters, integrated together with brachial blood pressure may allow to properly estimate non-invasively the PV loops at bedside.^75^ The need of dedicated software^76^ and the need of further validation studies currently limit the routine application of this approach in clinical practice. However, the development of non-invasive approach to extrapolate PV loops may considerably enhance our understanding of this phenomena and its clinical impact.

Infact, whether LV unloading is a necessary procedure or not, is still a debated argument in literature and day-to-day clinical practice. The current clinical practice reveals heterogeneity in timing (prophylactic or therapeutic), indications and techniques (pharmacological, interventional either surgical or percutaneous).^77^ As a consequence, LV unloading rate ranges from 2%^78^ to 68%.^79^ Although high quality evidence is still lacking in this field, upcoming registered clinical trials, named below, aiming to elucidate the role of LV unloading will be ready in the very next future. Protect IV (Impella-Supported PCI in High-Risk Patients With Complex Coronary Artery Disease and Reduced Left Ventricular Function; NCT04763200), inquires efficacy of Impella in patients undergoing high-risk PCI randomizing subjects to either Impella or standard care (with or without IABP). CHIP-BCIS3 (The Controlled Trial of High-Risk Coronary Intervention With Percutaneous Left Ventricular Unloading; NCT05003817) protocol takes into account PCI procedures in patients without cardiogenic shock, assessing elective LV unloading.

UNLOAD ECMO (Left Ventricular Unloading to Improve Outcome in Cardiogenic Shock Patients on V-A-ECMO; NCT04763200; NCT05577195) enlist patients on V-A-ECMO evaluating LV unloading during cardiocirculatory support, with a 1:1 randomization to treatment (V-A-ECMO + Impella) or control group (V-A-ECMO alone). Future results coming from randomized clinical trial will improve our knowledge on LV unloading and its application, leading to more solid evidence guided treatment.

## Limitations

Cardiogenic shock is a complex clinical syndrome which can be determined by right or left ventricle failure (or both). As matter of fact, first, this review only treated acute LV failures which lead to cardiogenic shock. Secondly, most of the data were obtained from observational studies, either monocentric or multicentric. In addition, no published multicentric randomized trial is available investigating the impact of LV unloading and the related strategies on the major clinical outcomes. Third, besides the aforementioned cardiovascular treatment, patients with severe cardiogenic shock often require meticulous multi-organ management (i.e. respiratory and coagulation) which was not the topic of this article. Finally, this review is a purposive (non-systematic) review and, therefore, does not use standardized methods for article selection and data extraction.

## Conclusions

Cardiogenic shock leads to a significant increase of EDP and EDV, as well as downward and rightward PV loop shifting. An effective LV unloading aims to minimize the myocardial oxygen consumption, namely reducing the PVA. Impella, IABP and Tandem Heart provide effective LV unloading. On the contrary, despite its ability to restore organs perfusion, extracorporeal life support in veno-arterial configuration may significantly overload the LV.

In acute setting, an effective LV unloading provides important benefits. Firstly, in case of ischemia, the related infarct size is significantly reduced. Secondly, the subsequent inflammation involving the myocardial tissue is limited and, therefore, the detrimental pathological cascade which ultimately leads to heart fibrosis and remodeling. Additionally, the concept of unloading may be broadly applied to other organs, such as the kidney. Further investigations are urgently needed to better define the role of organ unloading and its clinical implications.
